# Telemedicine adoption in Hungary: insights from two representative population surveys (2021–2024)

**DOI:** 10.1186/s12913-026-14262-2

**Published:** 2026-03-05

**Authors:** Bence Döbrössy, Edmond Girasek, Zsuzsa Győrffy

**Affiliations:** 1 Institute of Behavioural Sciences, Faculty of Medicine, Semmelweis University, 4 Nagyvárad tér. 20th floor, Budapest, 1089 Hungary; 2 Faculty of Health and Sport Sciences, Department of Psychology and Healthcare Management, Széchenyi István University, 1 Egyetem tér, Győr, 9026 Hungary; 3 Department of Family Medicine, Faculty of Medicine, Semmelweis University, 25, Üllői út, Budapest, 1091 Hungary

**Keywords:** Telemedicine, Population survey, Sociodemographic factors, Social support, Adoption

## Abstract

**Background:**

Despite increased interest, data on telemedicine adoption and patterns of use remain scarce. This study aims to fill this gap by analysing sociodemographic trends in telemedicine use in Hungary between 2021 and 2024.

**Methods:**

Two nationwide representative surveys were conducted in Hungary: a Computer Assisted Telephone Interview (CATI) with 1,500 participants in October 2021 and an online survey with 1,100 participants in February 2024. Both samples were stratified by gender, age, settlement type, and education. A Telemedicine Index, encompassing (a) online appointment booking and referral requests (b) video or phone teleconsultations (c) email communication with the doctor (d) sharing images with the doctor (e) sharing medical documentation with the doctor and (f) allowing the doctor to monitor changes in health status via smartphone was constructed. Statistical analyses included descriptive statistics, ANOVA, and multinomial logistic regression to assess sociodemographic factors associated with telemedicine use.

**Results:**

Telemedicine adoption increased between 2021 and 2024. The Telemedicine Index rose from Mean = 1.025 to Mean = 1.702. The proportion of non-users dropped from 43.5% to 21.0%. While initial disparities in 2021 showed women, younger individuals, higher educated people and urban residents using telemedicine more, these gaps narrowed significantly by 2024. Family support, indicated by living with a partner, positively influenced telemedicine adoption in both periods, emphasizing its role in facilitating use, especially among older adults and those with children. Analysis confirmed that education, age, gender, and family status are significantly associated with telemedicine use.

**Conclusion:**

Telemedicine has become increasingly integrated into Hungarian healthcare, with a shift from pandemic-driven necessity to broader societal adoption. While sociodemographic disparities persist, they have largely narrowed. The crucial role of social and family support in facilitating telemedicine use, particularly for vulnerable groups, highlights a key area for future interventions.

## Background

Telemedicine is defined as the remote delivery of healthcare using information and communication technologies [1]. It includes (a) telemonitoring – doctors monitoring patients’ health status remotely, (b) store and forward – delayed communication, via email or Electronic Health Record (EHR) between patient and provider and (c) teleconsultations – real time doctor-patient interactions via phone or video [2].

Although it was COVID-19 that accelerated telemedicine adoption [3–11], there is strong evidence of its benefit beyond the COVID-19 era [12]. International research shows that telemedicine can be used to address long standing health system issues including limited access to health care and staff shortages [13]. By making high quality healthcare available in marginalised populations, it can contribute towards health equity [14]. Results are very promising in chronic illness management [15]. There is evidence that high quality services can be provided through telemedicine. Reviews found that it reduces costs, and delivers comparable results to in-person care [16–18]. There is Hungarian research evidence, too. Virág et al. demonstrated that combining telemedicine with mobile health care units in developing regions can improve access to quality care [19].

There is emerging evidence of sociodemographic patterns in telemedicine use. Numerous studies can be found which identify disparities based on education, region, ethnicity, race income, age, and digital literacy. Results show that women, people with higher education, city dwellers and younger people are more likely to have used telemedicine [10, 20–22].

Research is still scarce on patient characteristics, type of telemedicine services and reasons for telemedicine use. Less than half of OECD countries, including Hungary, have this type of data [2]. The present study aims to fill this gap by assessing trends in telemedicine use between 2021 and 2024 and examining how sociodemographic factors are associated with its adoption in nationally representative samples of the Hungarian adult population. We are not aware of any other Hungarian or international survey-based studies that compare trends in telemedicine use during and after the pandemic on nationwide representative samples.

## Methods

This study is part of the research program “E-patients and E-physicians in Hungary: The Role and Opportunities of Digital Health Solutions in the Healthcare System” (OTKA-FK 134372), supported by the National Research, Development, and Innovation Office (NKFIH). Ethical approval was granted by the Medical Research Council – National Body, Hungary (IV-10927-1/EKU). Both surveys sampling and data-collection was carried out by Ipsos Zrt. Ipsos Zrt. is the Hungarian subsidiary of Ipsos, one of the world’s largest market research and public opinion companies. The firm conducts nationwide social, market, and opinion surveys using established methodological standards.

Two nationwide representative surveys were conducted. The first, in 2021, used computer-assisted telephone interviewing (CATI) with 1,500 adults. Stratified sampling ensured representation by gender, age, settlement type, and education. Data were collected between October 5–13. The sampling frame consisted of 12,000 individuals, selected randomly from an open telephone directory database, and an additional 8,000 individuals served as a reserve sample. A total of 11,733 respondents refused to participate, and 1,293 individuals dropped out, but most of this dropout occurred due to the sampling quota. During data collection, the reachability ratio was 80% mobile phone and 20% landline. We applied corrective weighting to the data to improve representativeness. The analysis was carried out with corrective weighting calculated to 1,500 individuals, so the sample size used in the analysis was 1,500.

To ensure representativeness the most recent national databases were used. In the case of the 2021 sample, this was the 2016 Microcensus [23]. A microcensus is a population survey conducted between two full censuses, usually halfway between them, using sample-based data collection to monitor social processes. Like the census, the microcensus is mandated by law. The Central Statistical Office carried out the Microcensus and about 10% of the population was surveyed. The same methodology is used as in a ‘real’ census, but on a smaller scale. In case of the 2024 sample the 2022 census was used to ensure representativeness [24].

The second survey, conducted in February 2024, used the same questionnaire but was administered online. The sample included 1,100 respondents: a 1,000-person nationally representative quota sample and a 100-person “senior boost” to better represent those aged 65+.

The survey was conducted using the Ipsos online panel. Due to business confidentiality, the size of the panel was not disclosed; however, the response rate among those invited ranged between 50% and 60% across different groups. The data collection continued until the number of respondents in each quota category reached the target specified in the sampling design matrix. For this reason, discussing a traditional response rate is less meaningful.

Here again the data were weighted by gender, age, education level, and settlement type, so these samples are representative of the Hungarian adult population. In both surveys data collection was completed within eight consecutive days (October 5–13 for 2021; March 8–15 for 2024), consistent with Ipsos’s standard rapid-fieldwork protocol for nationally representative omnibus surveys.

The same 25-item questionnaire was used in both surveys, with the only difference being the mode of administration. The average completion time was approximately 15 min, indicating good feasibility and low respondent burden. The questionnaire was developed specifically for this research project by the study team and has been previously used in a peer-reviewed study on older adults in the digital era, published in *BMC Geriatrics* in 2023, where the English version of the instrument is available [22].

The instrument comprises four main sections: (a) sociodemographic characteristics, (b) health status, (c) frequency and reasons for health-related internet use, and (d) knowledge, attitudes, and use of digital health technologies. As the present study focuses on sociodemographic trends in telemedicine use, not all sections of the questionnaire were analyzed.

Sociodemographic variables included age, gender, health status, residence type, family status, and number of children under 18 years. In addition, attitudes towards telemedicine solutions and self-reported telemedicine use were included in the analysis. As the analysed variables consisted primarily of sociodemographic characteristics and single-item measures of telemedicine use and attitudes, internal consistency metrics (e.g. Cronbach’s alpha) were not applicable. Item non-response for the analysed variables was low.

Next, the Telemedicine Index (TI) was constructed by summing the number of different telemedicine solutions used by each respondent, with higher values indicating a greater diversity of telemedicine engagement. The index captures six forms of remote health-care interaction aligned with the OECD concept of telemedicine: (1) online appointment booking, (2) remote consultations via telephone or video, (3) e-mail communication with a physician, (4) sharing medical images with a physician, (5) access to electronic medical records, and (6) telemonitoring [2]. 

Each component was coded as 0 (not used) or 1 (used in the previous 12 months), resulting in a composite score ranging from 0 to 6. For interpretative purposes, TI scores may be categorised as follows: 0 = no telemedicine use; 1–2 = low engagement (use of isolated or basic services); 3–4 = moderate engagement (use of multiple interactive services); and 5–6 = high engagement (broad and integrated use of telemedicine solutions). For certain analyses, the Telemedicine Index was dichotomised using a cut-off of 0 versus ≥ 1. A value of 0 indicates no use of any telemedicine solution, while values ≥ 1 indicate engagement with at least one form of telemedicine. This cut-off captures the fundamental distinction between non-users and users of telemedicine and has clear practical relevance from a public health and health-system perspective.

The Telemedicine Index demonstrated good internal consistency (Cronbach’s α = 0.78 in 2021 and 0.82 in 2024), supporting its use as a composite measure of telemedicine engagement.

### Statistical methods

Data analysis was conducted using IBM SPSS Statistics [25]. During statistical processing, we performed distribution analyses, chi-square tests, and analyses of variance (ANOVA). A significance level of 5% (*p* < 0.05) was applied when interpreting the results. For non-parametric comparisons, the Kruskal–Wallis test was used, and for two-group comparisons, the Mann–Whitney U test was applied.

To examine the constructed Telemedicine Index, multinomial logistic regression analysis was performed. The Telemedicine Index was categorized into three groups: non-users of telemedicine tools, users of up to two telemedicine tools, and users of at least three telemedicine tools.

## Results

Table 1 shows our results and the databases used to ensure representativeness. We can see that both samples are representative of the population in terms of age, gender, educational level and settlement type according to the most recent national census data.

**Table 1:** Demographic composition of the surveys compared to the 2016 microcensus and the 2022 population census

Category	Subcategory	Microcensus 2016		Our survey in 2021		Population census 2022		Our survey in 2024	
		%	n	%	n	%	n	%	n
Gender	Male	46,9%	3 794 810	46.6%	699	47,4%	3 755 336	47.4%	474
	Female	53,1%	4 291 984	53.4%	801	52,6%	4 163 500	52.6%	526
Age Group	18–29 years	17,6%	1 422 236	18.0%	270	16,0%	1 269 978	16.0%	160
	30–39 years	17,0%	1 378 176	19.7%	296	15,7%	1 239 557	15.7%	157
	40–49 years	18,8%	1 518 377	16.1%	242	19,9%	1 572 064	19.9%	199
	50–59 years	15,0%	1 217 038	17.8%	267	16,3%	1 292 541	16.3%	163
	60 years or older	31,5%	2 550 967	28.3%	425	32,1%	2 544 696	32.1%	321
Education Level	Less than high school diploma	45,4%	3 671 872	50.0%	750	43,5%	3 448 431	42.5%	425
	High school diploma	33,4%	2 699 261	32.0%	480	33,3%	2 636 689	34.3%	343
	Higher education degree	21,2%	1 715 661	18.0%	270	23,2%	1 833 716	23.2%	232
Settlement Type	Budapest	17,9%	1 447 536	18.1%	272	18,1%	1 432 306	18.4%	184
	County capital / city with county rights	18,6%	1 504 144	18.0%	270	20,8%	1 646 636	17.9%	179
	Other towns	37,4%	3 024 461	35.0%	525	32,0%	2 532 358	35.8%	358
	Villages / rural municipalities	26,1%	2 110 653	28.9%	434	29,1%	2 307 536	27.9%	279

Table 2. shows telemedicine use frequencies in 2021 and 2024. As can be seen the use of telemedicine solutions increased significantly between the two survey periods.

**Table 2:** Comparison of telemedicine use frequencies in 2021 and 2024

Service Type	2021		2024		
	%	*n*	%	*n*	*p*-value
Online appointment booking and referral requests	42.8%	642	69.8%	698	*p* < 0.001
Teleconsultation (by phone or video)	6.4%	96	14.2%	142	*p* < 0.001
Email communication with the doctor	24.0%	360	33.0%	330	*p* = 0.035
Sharing images with the doctor	8.1%	122	11.7%	117	Not significant
Sharing medical documentation with the doctor	18.9%	284	33.4%	334	*p* < 0.001
Doctor monitoring health status via smartphone	2.1%	32	7.5%	75	*p* = 0.032

All six examined areas experienced growth. Although teleconsultation use almost doubled, its adoption remains a relatively low 14%. In 2024, nearly 70% of patients booked appointments online, and over 30% communicated with their doctors via email and shared medical documents. In contrast, only about 10% of patients shared images or allowed doctors to monitor their health status through smartphones, indicating these practices were still uncommon.

The mean number of telemedicine solutions used in 2021 was 1.025 while in 2024 it was 1.702. This increase is even more evident if one looks at telemedicine frequencies in the two periods. This means that in 2024 more types of telemedicine solutions were used than in 2021. It does not mean however that it was used more frequently. The Telemedicine Index contains no information on the frequency different solutions were used, just on whether they were used at all.

Looking at Table 3. on telemedicine use frequencies; it is evident that there was a significant drop in the number of people not using any solutions and a rise among those using 2 or more. (According to Chi-square *p* < 0,001)

**Table 3:** Comparison of telemedicine index frequencies in 2021 and 2024

	2021		2024	
	%	*n*	%	*n*
0	43,5	653	21,0	210
1	28,7	431	30,1	301
2	15,4	231	25,2	252
3	7,6	113	12,6	126
4	3,7	55	5,7	57
5	1,1	16	2,9	29
6	0,0	0	2,4	24
Total	100,0	1500	100,0	1000

### Analysis of variance

To further explore the relationship between sociodemographic factors and telemedicine use, an analysis of mean differences was carried out using the F-statistic within an ANOVA framework. Although the Telemedicine Index did not follow a normal distribution, the sample size was sufficiently large to support the use of the parametric F-statistic. In addition to the parametric F-tests, non-parametric Mann–Whitney and Kruskal–Wallis p-values are also presented to assess the robustness of the results. Table 4 reports the ANOVA outcomes for 2021, while Table 5 presents the corresponding ANOVA results for 2024.

**Table 4:** Analysis of telemedicine index by sociodemographic subgroups in 2021, bold means *p* < 0.05

		Mean	*N*	Std. Deviation	F-test* p*-value	Mann-Whitney / Kruskall-Wallis test *p*-value
**Gender**	**Male**	**0,8913**	**699**	**1,11151**	***p*** ** < 0,001**	***p*** ** < 0,001**
	**Female**	**1,1409**	**801**	**1,23199**		
**Age groups**	**18-29 year old**	**1,2541**	**270**	**1,28325**	***p*** ** < 0,001**	***p*** ** < 0,001**
	**30-39 year old**	**1,2292**	**295**	**1,25121**		
	**40-49 year old**	**1,0550**	**242**	**1,19143**		
	**50-59** **year old**	**1,0990**	**267**	**1,18516**		
	**60 year old or more**	**0,6723**	**425**	**0,96916**		
**Level of education**	**No school leaving exam (trade school or less)**	**0,7144**	**750**	**0,98811**	***p*** ** < 0,001**	***p*** ** < 0,001**
	**Secondary school with school leaving exam**	**1,1945**	**480**	**1,23056**		
	**University or college**	**1,5849**	**270**	**1,32546**		
**Type of settlement**	**Budapest**	**1,2659**	**271**	**1,25432**	***p*** ** < 0,001**	***p*** ** < 0,001**
	**County seat**	**1,0974**	**270**	**1,22222**		
	**Town**	**1,0684**	**526**	**1,25949**		
	**Village**	**0,7756**	**434**	**0,95713**		
**Living alone or with partner**	**Lives alone**	**0,8992**	**610**	**1,13503**	***p*** ** < 0,001**	***p*** ** < 0,001**
	**Lives with a partner**	**1,1094**	**889**	**1,20936**		
**Number of children age less than 18 years**	**0**	**0,9375**	**1095**	**1,13096**	***p*** ** < 0,001**	***p*** ** < 0,001**
	**1**	**1,1795**	**185**	**1,24440**		
	**2**	**1,2505**	**156**	**1,27051**		
	**3 or more**	**1,5711**	**59**	**1,44992**		
Chronic disease	yes	1,0423	732	1,18860	*p*=0,589	*p*=0,536
	No	1,0092	765	1,18066		
**Labour market status**	**Active**	**1,1254**	**876**	**1,20829**	***p*** ** < 0,001**	***p*** ** < 0,001**
	**Non-active**	**0,8852**	**623**	**1,13444**		

Table 5. shows the results of the ANOVA analysis from 2024.

**Table 5:** Analysis of telemedicine index by sociodemographic subgroups in 2024, bold means *p* < 0.05

		Mean	*N*	Std. Deviation	F-test *p*-value	Mann-Whitney / Kruskall-Wallis test *p*-value
Gender	Male	1,6559	474	1,40657	*p* = 0,333	*p* = 0,337
	Female	1,7440	526	1,45857		
Age groups	18–29 year old	1,9493	160	1,51603	*p* = 0,065	*p* = 0,080
	30–39 year old	1,8324	157	1,60702		
	40–49 year old	1,6453	199	1,48900		
	50–59 year old	1,5711	163	1,38196		
	60 year old or more	1,6172	321	1,27447		
**Level of education**	**No school leaving exam (trade school or less)**	**1,4469**	**425**	**1,38813**	***p*** ** < 0,001**	***p*** ** < 0,001**
	**Secondary school with school leaving exam**	**1,8061**	**343**	**1,43990**		
	**University or college**	**2,0164**	**232**	**1,43287**		
**Type of settlement**	**Budapest**	**1,9338**	**184**	**1,36802**	***p*** ** = 0,002**	***p*** ** < 0,001**
	**County seat**	**1,9371**	**179**	**1,54542**		
	**Town**	**1,5748**	**358**	**1,41083**		
	**Village**	**1,5622**	**279**	**1,40068**		
**Living alone or with partner**	**Lives alone**	**1,4762**	**338**	**1,39783**	***p*** ** < 0,001**	***p*** ** < 0,001**
	**Lives with a partner**	**1,8194**	**660**	**1,44070**		
Number of children age less than 18 years	0	1,5564	590	1,29180	*p* = 0,001	*p* = 0,066
	1	1,8762	264	1,53042		
	2	2,0128	96	1,72475		
	3 or more	1,9075	51	1,69659		
**Chronic disease**	**yes**	**1,8217**	**568**	**1,40615**	***p*** ** = 0,004**	***p*** ** < 0,001**
	**No**	**1,5549**	**417**	**1,44890**		
**Labour market status**	**Active**	**1,8092**	**555**	**1,47120**	***p*** ** = 0,005**	***p*** ** = 0,004**
	**Non-active**	**1,5517**	**433**	**1,35539**		

In 2021, women were significantly more likely to use telemedicine services than men (Mean = 1.14 vs. 0.89, *p* < 0.001). By 2024, however, this gender disparity had disappeared, with no significant difference observed between the sexes (Mean = 1.66 vs. 1.74, *p* = 0.333).

In terms of age, 60 + adults reported considerably lower usage in 2021 (Mean = 0.672), while individuals under 40 showed higher engagement (Mean = 1.22 for the 30–39 age group, *p* < 0.001). By 2024, these age-related differences were no longer statistically significant, though there remained a slight trend toward greater use among younger people (Mean = 1.61 for the 60 + group and 1.83 for the 30–39 age group, *p* = 0.065). Telemedicine use among those over 60 years more than doubled. As for education level, there was more than a twofold difference between individuals with the lowest and highest educational attainment in 2021( Mean = 0.71 vs. 1.58, *p* < 0.001). Although this educational gap remained statistically significant in 2024 (Mean = 1.45 vs. 2.02, *p* < 0.001), it had narrowed.

In 2021, residents of smaller settlements used fewer telemedicine services (Mean = 0.78 in villages vs. 1.27 in Budapest, *p* < 0.001). By 2024, these geographical differences had lessened, though Budapest and county seats continued to show a significantly higher use (*p* < 0.002).

In 2021, individuals living with a partner made significantly greater use of telemedicine solutions than those living alone (Mean = 1.11 vs. 0.90). By 2024, this gap had widened further, with partnered individuals again showing higher usage (Mean = 1.82 vs. 1.48).

In 2021, households with children under 18 also showed notably higher telemedicine use. While people without children had an average usage of 0.94, those with three or more children reached a mean of 1.57. This pattern remained in 2024, although overall usage increased across all groups. However, according to the Mann–Whitney test, the differences were no longer statistically significant in 2024. The rise in telemedicine use was sharper among childless individuals than among those with children.

In 2021, telemedicine usage did not differ significantly between individuals with chronic illnesses (Mean = 1.04) and those without (Mean = 1.01). By 2024, however, people with chronic conditions were clearly using telemedicine more frequently (Mean = 1.82 vs. 1.55).

In both 2021 and 2024, individuals who were active in the labor market used significantly more telemedicine solutions than those who were inactive (2021: Mean = 1.13 vs. 0.89; 2024: Mean = 1.81 vs. 1.55). People engaged in any form of paid employment were classified as active, while full-time students, pensioners, unemployed individuals, and homemakers were classified as inactive.

To analyze the constructed Telemedicine Index, a multinomial logistic regression model was applied, with the Telemedicine Index serving as the dependent variable. In addition to socio-demographic variables—gender, age, type of settlement, highest educational attainment, and employment status—family situation (living alone or with a partner), number of children under 18, perceived benefits of digital health solutions, and presence of chronic illness were also included in the analysis. Because the index was not normally distributed, it was recoded into three categories: 0 (no use), 1–2 (moderate use), and 3 or more (high use), with the 0 category set as the reference group. For the 2021 data model, Nagelkerke R² is 0.177, which is considered acceptable in social science research.

In 2021, the following variables were significantly associated with telemedicine use at both moderate (index = 1–2) and high levels (index = 3+). Older individuals were less likely to engage in either moderate (OR = 0.984) or high use (OR = 0.968). Males were less likely than females to use telemedicine at moderate (OR = 0.752) or high levels (OR = 0.455). Chronic illness increased the likelihood of both moderate (OR = 1.569) and high use (OR = 2.095). The perceived advantages of digital health solutions were positively associated with high use (OR = 1.104). Family factors also played a role: living with more children under 18 was linked to higher odds of high telemedicine use (OR = 1.39), whereas place of residency influenced moderate use, with those living in Budapest more likely than village residents to engage at this level (OR = 1.48). Settlement type remained a strong predictor of high use, with residents of other towns (OR = 2.39), county seats (OR = 2.122), and Budapest (OR = 3.062) demonstrating significantly higher odds than those in villages. Educational attainment is also associated with usage patterns: individuals without a secondary school leaving exam were less likely to use telemedicine moderately (OR = 0.429), and for high use, both secondary education (OR = 0.432) and less than secondary education (OR = 0.178) were associated with substantially lower odds compared to those with college or university education.

The regression data for 2021 is shown in Table 6.

**Table 6:** Multinominal logistic regression analysis of telemedicine index categories by sociodemographic subgroups 2021

Multinominal logistic regression, Nagelkerke *R*-square = 0,177									
Telemedicine_index_3cat Telemedicine index 3 categories		B	Std. Error	Wald	df	Sig.	Exp(B)	95% Confidence Interval for Exp(B)	
								Lower Bound	Upper Bound
1–2	Intercept	1,064	0,393	7,315	1	0,007			
	Age	-0,016	0,004	16,474	1	0,000	0,984	0,976	0,992
	Gender: Male	-0,285	0,119	5,732	1	0,017	0,752	0,595	0,950
	Gender: Female	0^b^			0				
	How many advantages do digital health solutions have?	0,029	0,020	2,055	1	0,152	1,029	0,989	1,070
	How many disadvantages do digital health solutions have?	-0,007	0,023	0,109	1	0,741	0,993	0,950	1,037
	Number of children under 18	0,044	0,073	0,356	1	0,551	1,045	0,905	1,206
	Family status: lives alone	-0,201	0,122	2,685	1	0,101	0,818	0,644	1,040
	Family status: lives with a partner	0^b^			0				
	Do you have chronic illness: yes	0,450	0,128	12,414	1	0,000	1,569	1,221	2,016
	Do you have chronic illness: no	0^b^			0				
	Type of settlement: Budapest	0,392	0,181	4,684	1	0,030	1,480	1,038	2,110
	Type of settlement: county seat	0,022	0,174	0,016	1	0,901	1,022	0,727	1,437
	Type of settlement: town	-0,022	0,142	0,024	1	0,877	0,978	0,740	1,293
	Type of settlement: village	0^b^			0				
	Level of education: no secondary school leaving exam	-0,824	0,179	21,285	1	0,000	0,439	0,309	0,622
	Level of education: secondary school leaving exam	-0,348	0,186	3,521	1	0,061	0,706	0,491	1,016
	Level of education: college or university	0^b^			0				
	Labour market status: active	0,113	0,137	0,679	1	0,410	1,120	0,856	1,465
	Labour market status: inactive	0^b^			0				
3+	Intercept	-0,120	0,622	0,037	1	0,847			
	Age	-0,033	0,007	25,595	1	0,000	0,968	0,955	0,980
	Gender: Male	-0,787	0,192	16,812	1	0,000	0,455	0,312	0,663
	Gender: Female	0^b^			0				
	How many advantages do digital health solutions have?	0,099	0,037	7,128	1	0,008	1,104	1,027	1,188
	How many disadvantages do digital health solutions have?	-0,052	0,037	2,010	1	0,156	0,949	0,884	1,020
	Number of children under 18	0,329	0,095	11,878	1	0,001	1,390	1,152	1,676
	Family status: lives alone	-0,334	0,202	2,728	1	0,099	0,716	0,481	1,064
	Family status: lives with a partner	0^b^			0				
	Do you have chronic illness: yes	0,739	0,196	14,261	1	0,000	2,095	1,427	3,074
	Do you have chronic illness: no	0^b^			0				
	Type of settlement: Budapest	1,119	0,301	13,780	1	0,000	3,062	1,696	5,529
	Type of settlement: county seat	0,752	0,304	6,129	1	0,013	2,122	1,170	3,849
	Type of settlement: town	0,871	0,262	11,088	1	0,001	2,390	1,431	3,992
	Type of settlement: village	0^b^			0				
	Level of education: no secondary school leaving exam	-1,728	0,253	46,788	1	0,000	0,178	0,108	0,291
	Level of education: secondary school leaving exam	-0,839	0,238	12,413	1	0,000	0,432	0,271	0,689
	Level of education: college or university	0^b^			0				
	Labour market status: active	0,291	0,210	1,912	1	0,167	1,337	0,886	2,019
	Labour market status: inactive	0^b^			0				

a. The reference category is: ,00 0

b. This parameter is set to zero because it is redundant

A comparable multinomial logistic regression model was developed using the 2024 dataset. For this model Nagelkerke R² is 0.133, which is considered acceptable in social science research.

In 2024, several factors were significantly associated with both moderate (index = 1–2) and high telemedicine use (index = 3+). Living without a partner was linked to lower odds of both moderate (OR = 0.559) and high use (OR = 0.442). Chronic illness was positively associated with telemedicine engagement, increasing the likelihood of moderate use (OR = 1.553) and even more strongly for high use (OR = 2.370). Educational attainment remained a key determinant: compared to individuals with university or college education, those without a secondary school leaving exam were less likely to use telemedicine moderately (OR = 0.512), while those with less than secondary education had markedly lower odds of high use (OR = 0.234). Gender emerged as a predictor for high use, with males being less likely than females to use more telemedicine solutions (OR = 0.621). Perceived advantages of digital health were positively associated with high use (OR = 1.132), as was being economically active (OR = 1.583). Settlement type also influenced high telemedicine use, with residents of larger settlements showing higher odds compared to those in villages: county seats (OR = 2.133) and the capital city (OR = 2.379).

Table 7. shows the regression data for 2024.

**Table 7:** Multinominal logistic regression analysis of telemedicine index categories by sociodemographic subgroups in 2024

Multinominal logistic regression, Nagelkerke *R*-square = 0,133									
Telemedicine_index_3cat Telemedicine index 3 categories		B	Std. Error	Wald	df	Sig.	Exp(B)	95% Confidence Interval for Exp(B)	
								Lower Bound	Upper Bound
1–2	Intercept	0,845	0,495	2,917	1	0,088			
	Age	-0,001	0,006	0,046	1	0,830	0,999	0,987	1,011
	Gender: Male	0,043	0,024	3,086	1	0,079	1,044	0,995	1,095
	Gender: Female	0,025	0,027	0,863	1	0,353	1,026	0,972	1,082
	How many adavantages do digital health solutions have?	0,095	0,107	0,780	1	0,377	1,099	0,891	1,357
	How many disadavantages do digital health solutions have?	-0,299	0,183	2,659	1	0,103	0,742	0,518	1,062
	Number of children under 18	0^b^			0				
	family status: lives alone	-0,582	0,180	10,517	1	0,001	0,559	0,393	0,794
	Family status: lives with a partner	0^b^			0				
	Do you have chronic illness: yes	0,440	0,183	5,773	1	0,016	1,553	1,084	2,223
	Do you have chronic illness: no	0^b^			0				
	Type of settlement: Budapest	0,451	0,275	2,679	1	0,102	1,569	0,915	2,691
	Type of settlement: county seat	0,291	0,274	1,134	1	0,287	1,338	0,783	2,288
	Type of settlement: town	-0,099	0,208	0,225	1	0,635	0,906	0,603	1,362
	Type of settlement: village	0^b^			0				
	Level of education: no secondary school leaving exam	-0,670	0,240	7,808	1	0,005	0,512	0,320	0,819
	Level of education: secondary school leaving exam	0,068	0,257	0,071	1	0,790	1,071	0,647	1,771
	Level of education: college or university	0^b^			0				
	Labour market status active	0,177	0,188	0,881	1	0,348	1,193	0,825	1,726
	Labour market status inactive	0^b^			0				
3+	Intercept	-0,269	0,605	0,198	1	0,656			
	Age	-0,012	0,007	2,772	1	0,096	0,988	0,974	1,002
	Gender: male	0,124	0,032	15,126	1	0,000	1,132	1,063	1,205
	Gender: female	0,025	0,034	0,542	1	0,462	1,025	0,960	1,095
	How many advantages do digital health solutions have?	0,188	0,123	2,323	1	0,127	1,206	0,948	1,535
	How many disadavantages do digital health solutions have?	-0,477	0,220	4,688	1	0,030	0,621	0,403	0,956
	Number of children under 18	0^b^			0				
	family status: lives alone	-0,817	0,224	13,332	1	0,000	0,442	0,285	0,685
	Family status: lives with a partner	0^b^			0				
	Do you have chronic illness: yes	0,863	0,225	14,729	1	0,000	2,370	1,525	3,682
	Do you have chronic illness: no]	0^b^			0				
	Type of settlement: Budapest	0,867	0,325	7,093	1	0,008	2,379	1,257	4,502
	Type of settlement: county seat	0,757	0,323	5,488	1	0,019	2,133	1,132	4,019
	Type of settlement: town	0,088	0,262	0,114	1	0,736	1,092	0,654	1,825
	Type of settlement: village	0^b^			0				
	Level of education: no secondary school leaving exam	-1,452	0,280	26,909	1	0,000	0,234	0,135	0,405
	Level of education: secondary school leaving exam	-0,286	0,285	1,003	1	0,317	0,751	0,429	1,315
	Level of education: college or university	0^b^			0				
	Labour market status: active	0,459	0,231	3,958	1	0,047	1,583	1,007	2,489
	Labour market status: inactive	0^b^			0				

a. The reference category is: ,00 0

b. This parameter is set to zero because it is redundant

## Discussion

The use of telemedicine solutions increased between the two data collection periods. Online appointment booking increased the most from 42.8% to 69.8%. Sharing medical documents online went up from 18.9% to 33.4%. Although the use of teleconsultations also grew significantly (from 6.4% to 14.2%), its overall prevalence remains relatively low.

The Telemedicine Index Mean was significantly higher in 2024 than in 2021. The proportion of individuals not using any telemedicine (Telemedicine Index 0) halved in the period, while those using two or more telemedicine solutions more than doubled indicating a growing reliance on a diverse range of digital tools for accessing healthcare. This shift may be partly attributed to the widespread promotion of the ‘Egészségablak’ (healthwindow) app, which facilitates online appointment booking and provides streamlined access to EHR. The app is reported to have been downloaded 3.5 million times [26, 27].

Demographic differences in the use of telemedicine solutions decreased between 2021 and 2024. Gender differences were significant in 2021, with women demonstrating higher levels of engagement. These findings align with the international research [7, 21, 28]. By 2024, the gender gap had nearly disappeared. The rise in telemedicine use was more pronounced among men. Research attests that women visit primary healthcare more frequently than men [29]. This means that the digital and non-digital illness behaviour of men and women are different. Men are more active in the digital world when it comes to medical visits than in the real world.

Studies generally found that younger people used more telemedicine solutions [30–33]. In 2021, this was evident in the current research, too. Individuals over 60 used telemedicine significantly less than younger people. This changed by 2024. 60 + people had an accelerated increase in telemedicine use, so the age differences became insignificant. Interestingly, Hung et al.’s 2022 study found higher telemedicine use among the 80 + respondents than among the 18–29 age group. This may be due to the greatly increased health care needs of elderly people [6]. Haimi et al. (2024) looked at telemedicine use among Israelis aged 65 and older before, during, and after the COVID-19 pandemic [4]. It found that telemedicine use increased greatly during the period. Because elderly people are able to use it, telemedicine can be an alternative to nursing homes.

Although the gap narrowed, differences by educational attainment remained significant in 2024. Variance among people living in different settlement types also declined, though residents of Budapest and county seats still used more solutions. The observations that lower educated people and people living in rural areas used telemedicine less is very much supported by the literature.

Family support as indicated by living with a partner, was observed to have positively influenced the adoption of telemedicine services during both time periods. People living with a partner utilized a wider range of telemedicine options both in 2021 and 2024 than those living alone. A comparable trend was seen among individuals living with children under 18. A major contribution of this study is highlighting the role of social support as a beneficial factor in promoting telemedicine usage. Although this is a relatively unexplored area there is support for these findings [34]. In their 2021 US study on people aged 70 and over Chung et al. found that living with family or friends and being given technical support were associated with higher telemedicine utilization [35]. The significance of social support is further underscored by research conducted by the Digital Health Research Group at Semmelweis University, Hungary. Among individuals aged 65–74, 21.3% reported receiving help in finding health information online. This proportion increases to 35.4% among those aged 75 and older [22]. According to results published by Girasek et al. (2022) almost half of those who did not use the internet were helped by a friend or family in navigating digital health solutions [13]. As Győrffy et al. state in their 2023 analysis, the integration of seniors in the digital health era is vital [22]. Recognising that elderly individuals often need support when using the internet, the National Media and Infocommunications Authority launched the *Netre Fel!* initiative (a wordplay meaning “Ride the Net”). This program includes a guide specifically designed to help older adults navigate the internet. More importantly, it offers the option to request assistance online and connects users with volunteer “super-helpers” who provide personalized support [36].

Radó et al. give an example of the importance of peer support in telemedicine use among an other special needs population in their 2024 study [37]. The study identified a significantly digitally engaged subgroup among people experiencing homelessness. More than half of these digitally skilled individuals acted as informal digital supporters within their communities, assisting peers with problem-solving and basic digital literacy. This grassroots support network holds considerable potential for addressing the digital aspects of healthcare access among the homeless population.

Chronic illness became the strongest and most consistent predictor of telemedicine use, with chronically ill individuals showing significantly higher utilization in 2024 than in 2021. ANOVA results showed no significant difference in 2021 but by 2024 chronically ill individuals were using significantly more telemedicine solutions. In the regression analysis telemedicine use had strong association with having chronic illness in both years. In accordance with our results, many studies show a shift toward virtual care for managing chronic conditions. For example, Zaganjor et al. used the US 2022 National Health Interview Survey to look at telemedicine prevalence of the previous year among American adults with no prediabetes or diabetes diagnosis, diagnosed prediabetes, and diagnosed diabetes [38]. In 2021 and 2022, telemedicine use prevalence was 34.1% and 28.2% among adults without diagnosed diabetes or prediabetes, 47.6% and 37.6% among adults with prediabetes, and 52.8% and 39.4% among adults with diabetes, respectively. Telemedicine is widely recognized as a crucial tool for chronic disease management. A 2024 US study found that individuals with CVD had the highest odds of using any telemedicine when compared with those without CVD or CVD risk factors [39]. Perceptions of telemedicine also influenced use: although perceived benefits changed little between 2021 and 2024, perceived disadvantages decreased, making overall attitudes more favourable. Correlations between perceived advantages and higher Telemedicine Index scores strengthened over time, and in 2024 advantages were a significant predictor among high-level users. While telemedicine use in 2021 was often driven by necessity during the pandemic, by 2024 it increasingly reflected choice shaped by preferences and perceived value.

## Conclusion

Telemedicine use in Hungary significantly increased between 2021 (during the pandemic) and 2024 (post-pandemic), indicating its growing integration into everyday healthcare, driven by broader socio-cultural shifts rather than just pandemic necessity. Demographic disparities, including gender and age gaps, narrowed over this period, with increased use among men and the elderly. Social and family support emerged as a crucial positive factor influencing telemedicine use, particularly for older adults. The fact that in 2024 seeing advantages of telemedicine is a top determinant supports the belief that telemedicine is a driver of cultural and social transformation in health care [40]. It is this paradigm shift that needs to be promoted to help telemedicine achieve its full potential.

### Strengths, limitations

A key strength of the study is its use of two nationally representative surveys, enabling trend analysis of telemedicine adoption in Hungary from the pandemic to the post-pandemic period. The study also examines how sociodemographic factors influence telemedicine use and how these patterns evolved, offering valuable insights for targeted interventions.

However, the shift from telephone to an online survey could affect comparability. Differences in the mode of questionnaire administration may have led to differences in respondent compositions on the target variables between the modes. Nevertheless, literature suggests that two consecutive nationally representative repeated cross-sectional surveys can be meaningfully compared and are appropriate for trend estimation, provided they target the same population, employ similar sampling procedures and weighting, and use the same or equivalent questionnaires. One potential bias is internet use. Hungary’s internet penetration in early 2024 was 91.8%, while the online survey included 100% users. However, only 8% of the population were non-users, many of whom accessed online health information via family or friends, allowing indirect telemedicine use.

Socio-demographic characteristics were comparable across surveys, with stratified sampling and post-stratification weights ensuring alignment with census benchmarks. Identical questionnaires were used to minimize mode effects, and all questions were piloted for clarity. Telemedicine is not a sensitive topic, so social desirability bias is unlikely.

The cross-sectional design captures population-level patterns at two time points but not individual trajectories, and therefore limits causal inference.

Finally, while it maps usage patterns, the study does not assess the clinical effectiveness of telemedicine.

## Data Availability

The datasets used and/or analysed during the current study are available from the corresponding author on reasonable request.

## Abbreviations

WHO: World Health Organisation
OECD: Organization for economic cooperation and development
HER: Electronic health record
EU: European union
RSV: Relative search volume
ICT: Information and communication technology
COVID-19: Coronavirus disease 2019, a viral respiratory disease
HINTS: Health information national trends survey
CATI: Computer assisted telephone interview
ANOVA: Analysis of variance
IBM: International business machines corporation
